# Metabolic and Orexin-A Responses to Ketogenic Diet and Intermittent Fasting: A 12-Month Randomized Trial in Adults with Obesity

**DOI:** 10.3390/nu18020238

**Published:** 2026-01-12

**Authors:** Antonietta Monda, Maria Casillo, Salvatore Allocca, Fiorenzo Moscatelli, Marco La Marra, Vincenzo Monda, Girolamo Di Maio, Paride Vasco, Marcellino Monda, Rita Polito, Giovanni Messina, Antonietta Messina

**Affiliations:** 1 Department of Human Science and Quality of Life Promotion, San Raffaele Telematic University, 00166 Rome, Italy; antonietta.monda@uniroma5.it; 2 Section of Human Physiology, Unit of Dietetics and Sports Medicine, Department of Experimental Medicine, University of Campania “Luigi Vanvitelli”, 80138 Naples, Italy; maria.casillo@unicampania.it (M.C.); salvatore.allocca1@unicampania.it (S.A.); marco.lamarra@unicampania.it (M.L.M.); marcellino.monda@unicampania.it (M.M.); giovanni.messina@unicampania.it (G.M.); 3 Department of Education and Sport Sciences, Pegaso Telematic University, 80143 Naples, Italy; fiorenzo.moscatelli@unipegaso.it; 4 Department of Economics, Law, Cybersecurity, and Sports Sciences, University of Naples “Parthenope”, 80131 Naples, Italy; vincenzo.monda@uniparthenope.it; 5 Department of Psychology and Health Sciences, Pegaso Telematic University, 80143 Naples, Italy; girolamo.dimaio@unipegaso.it; 6 Department of Humanities, University of Foggia, 71100 Foggia, Italy; paride.vasco@unifg.it; 7 Department of Precision Medicine, University of Campania “Luigi Vanvitelli”, 80138 Naples, Italy; antonietta.messina@unicampania.it

**Keywords:** ketogenic diet, intermittent fasting, Orexin-A, metabolic flexibility, obesity management

## Abstract

Background/Objectives: Intermittent fasting and ketogenic dietary approaches are increasingly investigated for their potential metabolic benefits in obesity. However, their long-term neuroendocrine effects—particularly those involving Orexin-A, a peptide implicated in energy regulation—remain poorly understood. The objective of this study was to compare the long-term metabolic, inflammatory, and orexinergic responses to different dietary strategies in adults with obesity. Methods: In this 12-month randomized, three-arm trial, 30 adults with obesity (BMI ≥ 30 kg/m^2^) were randomly assigned (1:1:1) to a hypocaloric ketogenic diet (KD), a 16:8 time-restricted eating regimen (TRF16:8), or a 5:2 intermittent fasting protocol (ADF5:2). Anthropometric parameters, body composition, fasting glucose, lipid profile, inflammatory cytokines (CRP, IL-6, TNF-α, IL-10), and plasma Orexin-A levels were assessed at baseline and every 3 months. Dietary adherence was monitored through structured logs and monthly assessments. Statistical analyses included repeated-measures models with sensitivity analyses adjusted for age and sex. Results: All participants completed the intervention. The ketogenic diet produced the largest sustained reductions in BMI, fat mass, fasting glucose, and total cholesterol over 12 months. TRF16:8 elicited more rapid early metabolic improvements and showed the most consistent longitudinal increase in Orexin-A levels. The ADF5:2 protocol resulted in moderate improvements across outcomes. In all groups, increases in Orexin-A were associated with markers of improved metabolic flexibility and reduced inflammation; however, mediation analyses were exploratory and non-causal. Between-group differences remained significant for fat mass, glucose, and Orexin-A trajectories after correction for multiple comparisons. Conclusions: The ketogenic diet was associated with the most pronounced long-term metabolic improvements, whereas 16:8 time-restricted eating yielded faster early responses and the most stable enhancement in Orexin-A levels. These findings indicate distinct metabolic and neuroendocrine adaptation profiles across dietary strategies. Given the small sample size, results should be interpreted cautiously, and larger trials are warranted to clarify the role of Orexin-A as a potential biomarker of dietary response in obesity.

## 1. Introduction

Obesity represents one of the major global health challenges of the 21st century, being strongly associated with metabolic syndrome, insulin resistance, type 2 diabetes, cardiovascular disease, and a chronic low-grade inflammatory state [1]. The growing prevalence of obesity has prompted extensive investigation into efficacious, sustainable dietary strategies that can improve metabolic efficiency and mitigate the associated comorbidities. Among such strategies, both ketogenic diets (KD)—defined as very low-carbohydrate, high-fat dietary regimens that induce ketone body formation—and time-restricted feeding/intermittent fasting have emerged as promising approaches [2]. In humans, ketogenic dietary interventions have demonstrated reductions in visceral adipose tissue, improvements in glycemic and lipid profiles, and increased levels of the hypothalamic neuropeptide Orexin-A (also known as hypocretin-1) following intervention in obese subjects [3].

The orexinergic system—comprising orexin peptides (Orexin-A and Orexin-B) and their receptors (OX1R and OX2R)—is primarily known for its role in regulating arousal, wakefulness, and appetite. However, accumulating evidence reveals a more complex involvement in energy homeostasis, spontaneous physical activity, and metabolic adaptation. For instance, levels of plasma Orexin-A were found to be significantly lower in obese compared to lean individuals, suggesting its involvement in human energy metabolism and fat accumulation [4,5,6,7].

In experimental animal models, enhanced orexin signaling has been associated with increased spontaneous physical activity and resistance to diet-induced obesity. Moreover, orexin-A has been implicated in cardiovascular regulation, sympathetic activation, and the pathogenesis of obesity-related hypertension, underlining its integrative neuro-metabolic role [8,9].

In parallel, ketogenic diets and intermittent fasting regimens have been shown to modulate not only anthropometric and metabolic outcomes but also neuroendocrine signaling pathways. Nutritional ketosis leads to elevated ketone bodies (acetoacetate and β-hydroxybutyrate), which have been shown to serve as alternative energy substrates in colonocytes, maintain mucosal integrity, suppress inflammation, and carcinogenesis in the gut. Additionally, the ketogenic state may influence circadian rhythms and hypothalamic regulation of energy balance [10,11,12].

Despite these insights, the specific interplay between dietary interventions, inflammation, body composition, and orexinergic signaling remains insufficiently characterized in human obesity [13,14,15].

To address this gap, the present study aimed to evaluate the effects of three distinct dietary interventions—a ketogenic diet (KD), 16:8 time-restricted intermittent fasting (TRF), and alternate-day fasting (5:2)—on body composition, metabolic parameters, and plasma Orexin-A levels in obese participants over a 12-month period. We hypothesized that interventions that more effectively improve metabolic flexibility and reduce visceral adiposity will also lead to greater increases in plasma Orexin-A, thus reflecting neuro-metabolic adaptation. Findings from this work may provide novel insights into the neuroendocrine mechanisms underlying dietary modulation of obesity and support the implementation of orexin-directed biomarkers in nutritional interventions.

Intermittent fasting has been extensively investigated for its effects on weight loss, metabolic flexibility, and glycemic regulation. Moreover, recent research suggests that fasting-related metabolic adaptations may interact with orexinergic signaling, which is often reduced in individuals with obesity. However, long-term randomized comparisons of different fasting models with Orexin-A as a potential mediator are still lacking. To our knowledge, no previous study has directly compared a hypocaloric ketogenic-type diet, a 16:8 time-restricted eating regimen, and a 5:2 alternate-day fasting protocol over a 12-month period while evaluating Orexin-A trajectories. The present trial, therefore, addresses an important gap by providing the first long-term, three-arm randomized study assessing the metabolic and neuroendocrine responses to these interventions, and exploring whether changes in Orexin-A may partially mediate metabolic outcomes in adults with obesity.

## 2. Materials and Methods

### 2.1. Study Population

Thirty adults with obesity (15 males and 15 females), aged 20–40 years, were recruited from the Dietetics, Sports Medicine, and Psychophysical Well-being Unit of the University Hospital “Luigi Vanvitelli” (University of Campania), coordinated by Professor Marcellino Monda. During their initial clinical evaluation, participants were informed about three dietary approaches commonly used in routine nutritional care for obesity: a hypocaloric ketogenic-type diet (KD), a 16:8 time-restricted feeding pattern (TRF16:8), and a 5:2 alternate-day fasting regimen (ADF5:2).

Participants were then followed for 12 months with scheduled metabolic and hormonal assessments, without any experimental assignment or modification of usual care. The inclusion criteria were age 20–40 years; obesity defined according to WHO criteria as BMI ≥ 30 kg/m^2^; body composition was evaluated but not used as a selection parameter; stable body weight (±3 kg) over the preceding 3 months; and no participation in structured diet programs during the previous 6 months.

Exclusion criteria included: type 1 or type 2 diabetes, cardiovascular disease, endocrine disorders, pregnancy or breastfeeding, smoking, chronic inflammatory conditions, psychiatric illness, use of weight-loss medications, or any drug known to influence glucose or lipid metabolism. These criteria helped ensure a metabolically homogeneous sample. Medication use was reviewed at each visit; no participant initiated or discontinued medications capable of influencing metabolic outcomes during the observation period. All participants provided written informed consent before enrollment. The study was conducted according to the Declaration of Helsinki and approved by the Ethics Committee of the University of Campania “Luigi Vanvitelli” (protocol 0003232/1, 1 February 2023).

### 2.2. Study Design

This study was conducted as a 12-month, non-interventional, observational comparison of three commonly adopted dietary approaches used in routine nutritional counseling for obesity: a hypocaloric ketogenic-type diet (KD), a 16:8 time-restricted feeding pattern (TRF16:8), and a 5:2 alternate-day fasting regimen (ADF5:2).

After group selection, individuals were monitored from baseline (T0) to 12 months (T3) with repeated assessments of metabolic and hormonal parameters. No randomization, allocation procedures, or concealed assignment were used, as the study did not seek to test a therapeutic intervention but rather to observe naturally occurring differences among participants following dietary models already established in clinical practice.

The research team did not alter participants’ usual care, did not prescribe interventions beyond standard dietary advice, and did not introduce any experimental treatment. All procedures adhered to a non-interventional design, focusing solely on longitudinal monitoring of outcomes.

### 2.3. Dietary Interventions

Three dietary interventions were implemented to compare their impact on metabolic efficiency, inflammation, and the prevention of obesity-related metabolic impairments.

The Ketogenic Diet (KD) consisted of a daily caloric intake of approximately 1200 kcal, with a very low carbohydrate allowance (≤50 g/day). The diet was primarily composed of fats (70–75%) and moderate protein (20–25%). This macronutrient profile was designed to induce and maintain nutritional ketosis throughout the intervention period. The KD group followed a standardized hypocaloric prescription to ensure induction of nutritional ketosis. We acknowledge that caloric requirements vary individually; however, this standardized restriction is consistent with clinical ketogenic protocols used in obesity management. Dietary adherence was monitored each day in the early afternoon (between 2:00 and 4:00 p.m.) by measuring capillary blood ketone concentrations using GD40 Delta test strips (TaiDoc Technology Co., New Taipei City, Taiwan). A state of nutritional ketosis was considered present when β-hydroxybutyrate values exceeded 0.5 mmol/L.

**The Intermittent Fasting 16:8 protocol** required participants to fast for 16 consecutive hours each day, limiting food intake to an 8-h eating window (for example, from 12:00 p.m. to 8:00 p.m.). Total daily caloric intake was comparable to that of the ketogenic diet but redistributed within the restricted feeding window. The dietary composition during the eating period included 40–45% carbohydrates, 30–35% fats—mainly from mono- and polyunsaturated sources—and 20–25% proteins. Water, black coffee, and unsweetened tea were permitted during fasting hours. This time-restricted pattern enhances metabolic flexibility by alternating prolonged fasting with controlled nutrient intake, thereby supporting improvements in fat utilization and insulin sensitivity.

**The Intermittent Fasting 5:2 regimen** involved two non-consecutive fasting days per week (e.g., Monday and Thursday), during which participants consumed only 20–25% of their daily energy requirements (approximately 500–600 kcal/day). On five fasting days, macronutrient distribution was set at 30% carbohydrates, 45% fats—mainly omega-3 and other unsaturated fatty acids—and 25% proteins. Food choices included lean protein sources, vegetables, low-glycemic fruits, and small amounts of healthy fats. During the remaining five days, participants ate ad libitum. This approach emulates intermittent caloric restriction while maintaining weekly dietary flexibility, a structure known to facilitate compliance and promote metabolic improvements.

### 2.4. Monitoring and Measurements

Assessments were conducted at baseline (T0) and repeated at regular intervals (T1, T2, and T3) over a total period of 12 months.

The following parameters were collected:

#### 2.4.1. Anthropometric Measurements

- Weight and height were measured with standardized instruments.
- Body Mass Index (BMI) was calculated as the weight/height ratio^2^ (kg/m^2^).
- Body composition by Bioimpedance Analysis (BIA): the assessment was performed with a multifrequency analyzer (BIA 101 Anniversary, Akern Srl, Florence, Italy) under standardized conditions (fasting for at least 8 h, empty bladder, and avoiding intense physical activity in the previous 24 h). BIA allows for the estimation of fat mass, lean mass, total body water, and intracellular and extracellular compartments, providing a detailed picture of body composition.

Dietary adherence was monitored through weekly diet logs and structured dietary recalls at each visit. Trends in body composition (BIA) and metabolic parameters were also used as indirect adherence indicators.

#### 2.4.2. Blood Chemistry Parameters

- Venous blood samples were drawn in the morning after fasting for at least 8 h, collected in EDTA-anticoagulated tubes. Plasma was obtained by centrifugation at 3000 rpm for 15 min at 4 °C, aliquoted, and stored at −80 °C until analysis.
- Lipid profile analysis (total cholesterol, HDL, LDL, triglycerides) and glycemic profile. Serum levels of Orexin-A were determined using an Enzyme-Linked Immunosorbent Assay (ELISA), using commercial kits specific for each marker (R&D Systems, Minneapolis, MN, USA; Thermo Fisher Scientific, Waltham, MA, USA).

### 2.5. Statistical Analysis

All statistical analyses were performed using IBM SPSS Statistics software (version 28.0; IBM Corp., Armonk, NY, USA) and R software (version 4.3.2; R Foundation for Statistical Computing, Vienna, Austria). Graphical representations were generated with GraphPad Prism (version 10.1; GraphPad Software, San Diego, CA, USA). Continuous variables were expressed as mean ± standard deviation (SD). The Shapiro–Wilk test was used to assess normality of the distributions, and Levene’s test to verify homogeneity of variances. When the assumption of sphericity was violated, the Greenhouse–Geisser correction was applied to adjust the degrees of freedom. The level of statistical significance was set at *p* < 0.05 (two-tailed) for all tests.

Baseline comparability among intervention groups was assessed using one-way ANOVA (or Kruskal–Wallis test when distributional assumptions were not met) for continuous variables and χ^2^ or Fisher’s exact tests for categorical variables; baseline descriptive statistics and between-group comparisons are reported in Table 1.

A two-way repeated-measures analysis of variance (RM-ANOVA) was used to evaluate the effects of time (T0, T1, T2, T3; within-subject factor), dietary intervention (ketogenic diet, intermittent fasting 16:8, alternate-day fasting 5:2; between-subject factor), and their interaction (time × group) on anthropometric, biochemical, and hormonal parameters. When significant main or interaction effects were detected, Tukey’s honestly significant difference (HSD) post hoc tests were conducted with adjustment for multiple comparisons. Post hoc analyses were used to determine (i) within-group differences over time compared with baseline (T0) and (ii) between-group differences at each time point (T0, T1, T2, T3) based on pairwise group contrasts within each time level. For each RM-ANOVA model, F values, degrees of freedom (df), *p* values, and partial eta-squared (η^2^) were reported to quantify effect size and the proportion of variance explained by each factor. Effect sizes were interpreted according to Cohen’s criteria (small ≥ 0.01, medium ≥ 0.06, large ≥ 0.14).

Given the number of repeated-measures analyses conducted across multiple outcomes, we acknowledge the potential for inflation of type I error when interpreting findings across endpoints. To mitigate this risk and enhance interpretability, outcomes were interpreted with a hierarchical emphasis: primary/clinically relevant endpoints (BMI, fat mass, fasting glucose, total cholesterol, and Orexin-A) were prioritized for inference, whereas remaining biochemical and inflammatory markers were considered secondary/exploratory and interpreted cautiously in the context of biological plausibility and consistency across analyses. Tukey-adjusted post hoc comparisons were used to control multiplicity within each outcome for pairwise contrasts.

To explore the relationships between metabolic and neuroendocrine adaptations, Pearson’s correlation coefficients (r) were calculated between changes (ΔT3 − T0) in Orexin-A levels and variations in BMI, fat mass, glucose, and total cholesterol; in the presence of non-normal distributions, Spearman’s rank correlations (ρ) were used. Furthermore, to assess whether changes in Orexin-A mediated the effects of dietary intervention on metabolic outcomes, hierarchical linear regression and mediation analyses were performed using the PROCESS macro (Model 4) for SPSS, with dietary group as the independent variable, ΔOrexin-A as the mediator, and ΔBMI or Δglucose as dependent variables. Indirect effects were tested using bootstrapping procedures (5000 samples) to obtain bias-corrected 95% confidence intervals (CIs). Model fit was evaluated using the coefficient of determination (R^2^), and variance inflation factors (VIF < 2) were inspected to exclude multicollinearity. All analyses were conducted under a two-sided hypothesis-testing framework, and results were visualized as means ± SD or scatter plots with regression lines illustrating associations between Orexin-A and metabolic parameters.

To assess the robustness of the primary findings and to obtain adjusted estimates, additional sensitivity analyses adjusted for age and sex were performed using linear mixed-effects models. For each outcome, models included group, time, and the group × time interaction as fixed effects, with age and sex entered as covariates, and a random intercept for participant to account for within-subject correlation across repeated measures. Adjusted estimated marginal means and group × time contrasts with 95% confidence intervals were derived from these models.

## 3. Results

Baseline demographic, anthropometric, metabolic, and neuroendocrine characteristics were comparable across the three dietary intervention groups (Table 1). No statistically significant differences were observed at baseline for BMI, fat mass, fasting glucose, total cholesterol, or circulating Orexin-A levels, indicating a similar degree of obesity, metabolic status, and neuroendocrine profile at study entry. These findings confirm adequate baseline comparability among groups prior to the initiation of the dietary interventions (Table 1).

**Figure 1:**
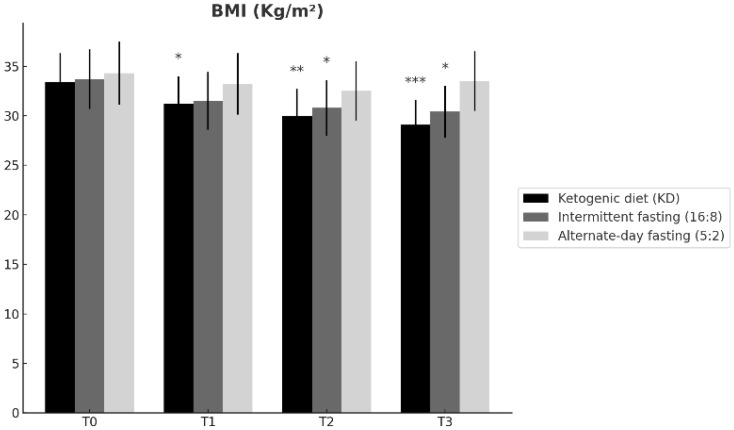
Changes in body mass index (BMI) over time (T0, T1, T2, T3) in participants following the ketogenic diet (KD), time-restricted feeding 16:8 (TRF 16:8), and alternate-day fasting 5:2 (ADF 5:2). Data are presented as mean ± SD. Statistical significance vs. baseline (T0) within the same group is indicated as * *p* < 0.05, ** *p* < 0.01, *** *p* < 0.001.

Baseline BMI was comparable across groups (Table 1). BMI changed differentially over time across the dietary interventions (time × group interaction; Table 2). From T0 to T3, the ketogenic diet (KD) produced the largest reduction (33.4 ± 2.9 to 29.1 ± 2.5 kg/m^2^; Δ = −4.3), followed by time-restricted feeding 16:8 (33.7 ± 3.0 to 30.4 ± 2.6 kg/m^2^; Δ = −3.3), whereas alternate-day fasting 5:2 showed a smaller change (34.3 ± 3.2 to 33.5 ± 3.0 kg/m^2^; Δ = −0.8). At T3, BMI was significantly lower in KD than in ADF 5:2 (Tukey-adjusted *p* = 0.004; Table 2), while the difference between KD and TRF 16:8 was not significant (*p* = 0.11). Overall, these results indicate that KD and TRF 16:8 improved BMI over 12 months, with KD showing the most pronounced reduction (Figure 1).

**Figure 2:**
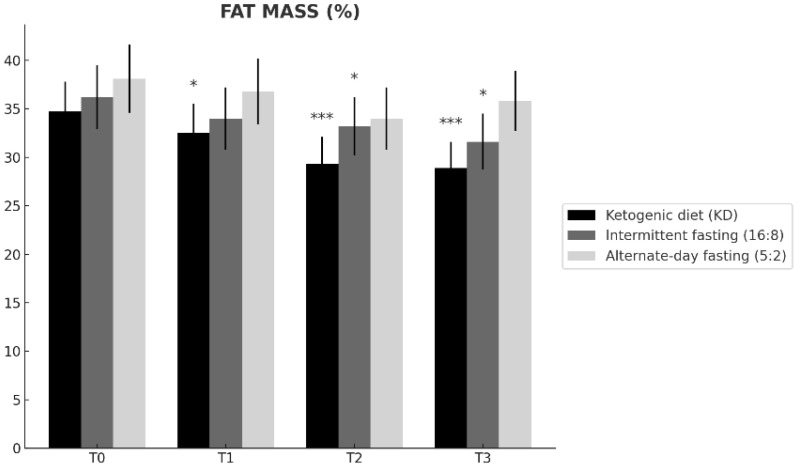
Changes in fat mass over time (T0, T1, T2, T3) in the ketogenic diet (KD), time-restricted feeding 16:8 (TRF 16:8), and alternate-day fasting 5:2 (ADF 5:2) groups. Values are expressed as mean ± SD. Statistical significance vs. baseline (T0) within each group is indicated as * *p* < 0.05, *** *p* < 0.001.

Baseline fat mass was comparable across groups (Table 1). Fat mass changed differentially over time across the dietary interventions (time × group interaction; Table 2). From T0 to T3, the ketogenic diet (KD) showed the largest reduction (34.7 ± 3.1% to 28.9 ± 2.7%; Δ = −5.8), followed by time-restricted feeding 16:8 (36.2 ± 3.3% to 31.6 ± 2.9%; Δ = −4.6), whereas alternate-day fasting 5:2 exhibited a smaller change (38.1 ± 3.5% to 35.8 ± 3.1%; Δ = −2.3). At T3, fat mass was significantly lower in KD than in ADF 5:2 (Tukey-adjusted *p* = 0.006; Table 2), while the difference between KD and TRF 16:8 was not significant (*p* = 0.14). Overall, these findings indicate that KD and TRF 16:8 improved adiposity over 12 months, with KD producing the most pronounced reduction (Figure 2).

**Figure 3:**
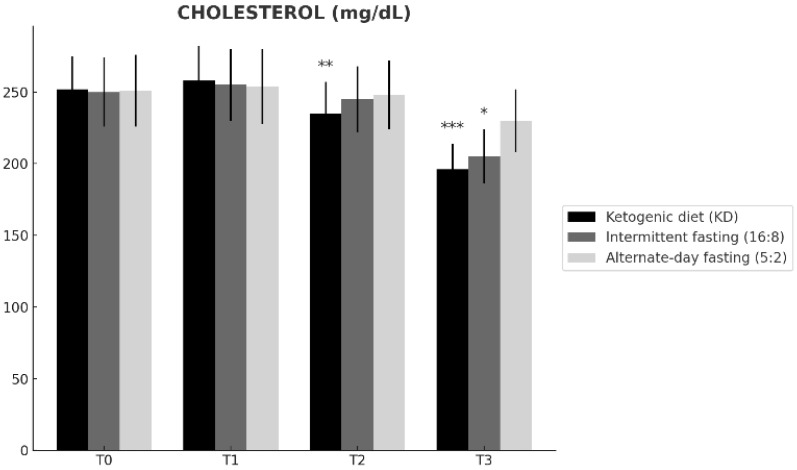
Changes in total cholesterol levels over time (T0–T3) in participants assigned to the ketogenic diet (KD), time-restricted feeding 16:8 (TRF 16:8), and alternate-day fasting 5:2 (ADF 5:2). Data are presented as mean ± SD. Within-group differences versus baseline (T0) are denoted as * *p* < 0.05, ** *p* < 0.01, *** *p* < 0.001.

Baseline total cholesterol was comparable across groups (Table 1). Total cholesterol changed differentially over time across the dietary interventions (time × group interaction; Table 2). From T0 to T3, the ketogenic diet (KD) showed the largest reduction (252 ± 23 to 196 ± 18 mg/dL; Δ = −56), followed by time-restricted feeding 16:8 (250 ± 24 to 205 ± 19 mg/dL; Δ = −45), whereas alternate-day fasting 5:2 exhibited a smaller change (251 ± 25 to 230 ± 22 mg/dL; Δ = −21). At T3, total cholesterol was significantly lower in KD than in ADF 5:2 (Tukey-adjusted *p* = 0.008; Table 2), while the difference between KD and TRF 16:8 was not significant (*p* = 0.16). Overall, these findings indicate a stronger lipid-lowering effect of KD over 12 months (Figure 3).

**Figure 4:**
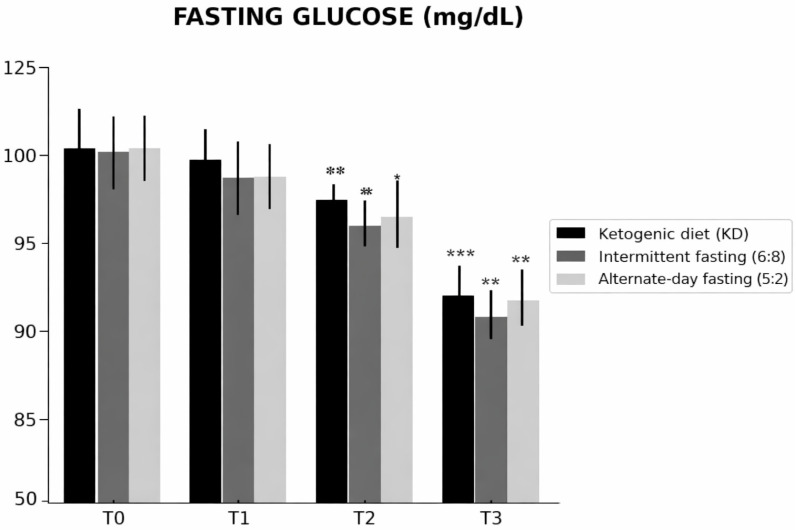
Changes in fasting glucose levels over time (T0–T3) in participants assigned to the ketogenic diet (KD), time-restricted feeding 16:8 (TRF 16:8), and alternate-day fasting 5:2 (ADF 5:2). Data are presented as mean ± SD. Within-group differences versus baseline (T0) are indicated as * *p* < 0.05, ** *p* < 0.01, *** *p* < 0.001. Fasting glucose levels decreased significantly over time in the ketogenic diet and time-restricted feeding 16:8 groups, whereas the alternate-day fasting 5:2 regimen was associated with smaller and non-significant changes.

Baseline fasting glucose was comparable across groups (Table 1). Fasting glucose changed differentially over time across the dietary interventions (time × group interaction; Table 2). From T0 to T3, the ketogenic diet (KD) showed the largest reduction (108 ± 8 to 87 ± 6 mg/dL; Δ = −21), followed by time-restricted feeding 16:8 (110 ± 9 to 95 ± 7 mg/dL; Δ = −15), whereas alternate-day fasting 5:2 exhibited a smaller change (112 ± 9 to 106 ± 8 mg/dL; Δ = −6). At T3, fasting glucose was significantly lower in KD than in ADF 5:2 (Tukey-adjusted *p* = 0.007; Table 2), while the difference between KD and TRF 16:8 was not significant (Table 2). Overall, these findings indicate a stronger glucose-lowering effect of KD over 12 months (Figure 4).

**Figure 5:**
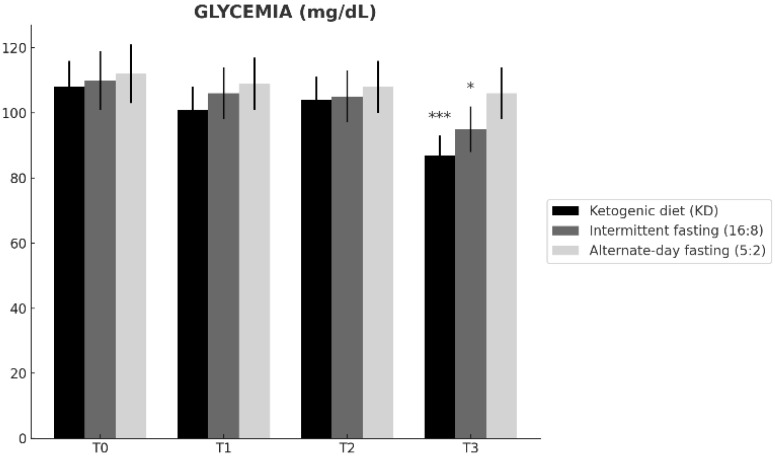
Changes in circulating Orexin-A concentrations over time (T0–T3) in the ketogenic diet (KD), time-restricted feeding 16:8 (TRF 16:8), and alternate-day fasting 5:2 (ADF 5:2) groups. Values are presented as mean ± SD. Within-group differences compared with baseline (T0) are indicated as * *p* < 0.05, *** *p* < 0.001.

Baseline Orexin-A levels were comparable across groups (Table 1). Orexin-A changed differentially over time across the dietary interventions (time × group interaction; Table 2). From T0 to T3, the ketogenic diet (KD) showed the largest increase (2.1 ± 0.3 to 3.4 ± 0.4 ng/mL; Δ = +1.3), followed by time-restricted feeding 16:8 (2.0 ± 0.3 to 3.0 ± 0.4 ng/mL; Δ = +1.0), whereas alternate-day fasting 5:2 exhibited a smaller change (2.1 ± 0.3 to 2.6 ± 0.3 ng/mL; Δ = +0.5). At T3, Orexin-A levels were significantly higher in KD than in ADF 5:2 (Tukey-adjusted *p* = 0.006; Table 2), while the difference between KD and TRF 16:8 was not significant (*p* = 0.12). Overall, these findings indicate a stronger orexinergic response to KD over 12 months (Figure 5).

Tukey-adjusted pairwise comparisons between dietary groups at each time point are summarized in Table 1. No significant between-group differences were observed at baseline (T0) or during the early phases of the intervention (T1 and T2) for any of the analyzed outcomes. In contrast, at the end of the intervention (T3), the ketogenic diet (KD) showed significantly greater changes compared with the alternate-day fasting 5:2 (ADF 5:2) group for BMI, fat mass, fasting glucose, total cholesterol, and Orexin-A, whereas no significant differences were detected between KD and time-restricted feeding 16:8 (TRF 16:8) for anthropometric outcomes.

A two-way repeated-measures ANOVA was conducted to assess the effects of time, dietary intervention, and their interaction on anthropometric, biochemical, and hormonal parameters. As summarized in Table 3, all outcomes exhibited a significant main effect of time (*p* < 0.001), indicating progressive improvements throughout the 12-month intervention. A significant time × group interaction was also observed for every variable (*p* ≤ 0.01), demonstrating that the magnitude and temporal pattern of change differed across dietary regimens. Across all outcomes, the Ketogenic diet (KD) elicited the most pronounced and consistent adaptations, followed by Intermittent fasting (16:8), whereas the Alternate-day fasting (5:2) protocol produced smaller or delayed effects. Effect sizes were generally large (η^2^ = 0.41–0.50), and the statistical models explained 45–52% of total variance (R^2^ = 0.45–0.52), supporting the robustness of these findings. In sensitivity analyses, age- and sex-adjusted linear mixed-effects models confirmed the primary findings, with the group × time interaction remaining statistically significant for the main outcomes. Participants following the Ketogenic diet showed the greatest reductions in BMI, fat mass, glucose, and total cholesterol, along with a marked increase in Orexin-A levels. Intermittent fasting (16:8) induced similar but slightly less pronounced improvements, while the 5:2 regimen resulted in limited changes, particularly in body composition and metabolic control. These results indicate that both the Ketogenic diet and Intermittent fasting (16:8) were effective in modulating metabolic and neuroendocrine parameters, with the Ketogenic diet emerging as the most potent and sustained intervention (Table 3).

As summarized in Table 4, all dietary interventions were associated with improvements in anthropometric, metabolic, and neuroendocrine parameters over the 12-month study period, with clear differences in magnitude across regimens. The ketogenic diet (KD) produced the most pronounced changes, showing the largest reductions in BMI (ΔT3 − T0 = −4.3 kg/m^2^), fat mass (−5.8%), fasting glucose (−21 mg/dL), and total cholesterol (−56 mg/dL), together with the greatest increase in circulating Orexin-A levels (+1.3 ng/mL). Time-restricted feeding 16:8 also resulted in meaningful improvements across outcomes, although of smaller magnitude than KD, whereas the alternate-day fasting 5:2 regimen was associated with more modest or non-significant changes. Overall, these end-of-study data confirm a gradient of effectiveness across dietary interventions, with the ketogenic diet eliciting the strongest and most consistent metabolic and neuroendocrine adaptations.

To further explore the neuro-metabolic mechanisms underlying the dietary interventions, correlation and mediation analyses were performed between changes in Orexin-A (ΔT3 − T0) and variations in the main metabolic outcomes. A strong inverse correlation was found between ΔOrexin-A and ΔBMI (*r* = −0.62, *p* < 0.001) as well as Δfat mass (*r* = −0.58, *p* < 0.001), indicating that larger increases in Orexin-A were associated with greater reductions in adiposity. Similarly, ΔOrexin-A was negatively correlated with Δglucose (*r* = −0.54, *p* = 0.002) and Δtotal cholesterol (*r* = −0.49, *p* = 0.005), suggesting an overall enhancement of metabolic flexibility and glycemic control in subjects showing stronger orexinergic activation. A positive correlation was also observed between ΔOrexin-A and Δlean mass (*r* = +0.46, *p* = 0.007), further supporting a beneficial relationship between neuroendocrine regulation and preservation of metabolically active tissue. When correlations were analyzed separately by dietary group, the Ketogenic diet and Intermittent fasting (16:8) groups displayed the strongest associations (|r| range 0.55−0.70, *p* < 0.01), whereas weaker or non-significant correlations were found in the 5:2 fasting groups (*p* > 0.05). To assess whether Orexin-A modulated the effects of diet on metabolic outcomes, hierarchical linear regression analyses were conducted with dietary regimen as the independent variable, ΔOrexin-A as the mediator, and ΔBMI or Δglucose as dependent variables. In the full model, dietary intervention significantly predicted changes in BMI (*β* = −0.53, *p* < 0.001) and glucose (*β* = −0.48, *p* = 0.002). When ΔOrexin-A was added as a mediator, the predictive effect of diet decreased (*β* = −0.32, *p* = 0.01 for BMI; *β* = −0.28, *p* = 0.03 for glucose), while ΔOrexin-A remained a significant independent predictor (*β* = −0.38, *p* = 0.004 for BMI; *β* = −0.35, *p* = 0.006 for glucose). The indirect effect of diet through ΔOrexin-A was statistically significant (95% CI [−0.29 to −0.08]), accounting for approximately 35% of the total variance in BMI reduction and 30% of glucose improvement, thus confirming a partial mediation effect of Orexin-A on the metabolic benefits of dietary intervention. Collectively, these results suggest that the upregulation of Orexin-A plays a central role in enhancing metabolic flexibility, promoting fat utilization, and improving glucose and lipid homeostasis.

**Figure 6:**
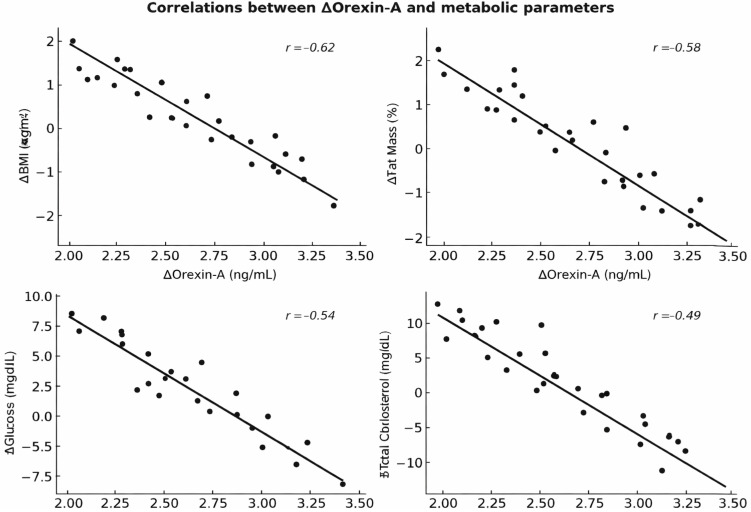
Correlations between changes in Orexin-A (ΔOrexin-A) and metabolic parameters (ΔBMI, ΔFat Mass, ΔGlucose, and ΔTotal Cholesterol) across the 12-month intervention period. Each scatter plot shows a significant inverse association between ΔOrexin-A and the corresponding metabolic variable, indicating that higher increases in Orexin-A were associated with greater improvements in body composition and metabolic control. Data are presented as individual values with linear regression lines (black) and Pearson’s correlation coefficients (italicized). Statistical significance was set at *p* < 0.05.

Such findings support the hypothesis that neuroendocrine adaptation via Orexin-A may represent a key biological pathway linking time-restricted feeding and ketogenic metabolism to improved metabolic health in obesity (Figure 6).

## 4. Discussion

The present study evaluated the long-term effects of three dietary strategies—Ketogenic Diet (KD), Time-Restricted Intermittent Fasting (16:8), and Alternate-Day Fasting (5:2)—on metabolic and neuroendocrine adaptations in obese adults. Overall, the findings demonstrate that both KD and 16:8 fasting elicited significant improvements in body composition and biochemical parameters, accompanied by a marked rise in circulating Orexin-A levels. Conversely, the 5:2 fasting regimen induced only modest, non-significant changes across most variables. These results highlight substantial differences in the metabolic impact of distinct dietary strategies and point toward Orexin-A as a potential biomarker mediating neuro-metabolic adaptation to sustained dietary interventions. A key observation of this study is the great improvement in anthropometric parameters under KD and 16:8 fasting, including significant reductions in BMI and fat mass, paralleled by increases in lean body mass. These findings are consistent with previous research demonstrating the efficacy of time-restricted feeding and ketogenic nutrition in reducing adiposity and improving energy metabolism through enhanced fat oxidation and reduced insulin levels [16,17,18]. Importantly, our correlation analyses revealed a robust inverse relationship between changes in Orexin-A and both BMI (r = −0.62) and fat mass (r = −0.58), suggesting that individuals experiencing greater orexinergic activation also exhibited more pronounced improvements in overall body composition. This aligns with evidence indicating lower Orexin-A levels in obese individuals and supports the hypothesis that restoring orexinergic tone may facilitate weight reduction and fat mobilization [19,20,21,22].

From a biochemical perspective, significant reductions in fasting glucose and total cholesterol were observed in the KD and 16:8 groups, consistent with improved glycemic control and lipid utilization. These metabolic shifts were strongly correlated with increases in Orexin-A, which showed negative associations with Δglucose (r = −0.54) and Δtotal cholesterol (r = −0.49). Notably, Orexin-A is known to stimulate sympathetic activity, enhance spontaneous physical activity, and increase non-exercise thermogenesis—mechanisms that collectively improve metabolic flexibility and substrate utilization [23,24,25]. The observed correlations reinforce the concept that Orexin-A may serve as an endocrine integrator promoting the transition toward more efficient metabolic states under nutritional interventions.

Furthermore, mediation analyses indicated that changes in Orexin-A partially accounted for the effects of dietary intervention on BMI and glucose, with indirect effects explaining approximately 35% of the variance in BMI reduction and 30% of the improvement in fasting glucose. This suggests that Orexin-A is not merely a biomarker of metabolic improvement but may actively contribute to the beneficial outcomes observed. The ketogenic state, characterized by elevated β-hydroxybutyrate levels, may further augment orexinergic signaling by modulating hypothalamic energy sensors and stabilizing neuronal excitability—thus enhancing metabolic efficiency and reducing inflammatory responses. This is consistent with reports that ketone bodies support anti-inflammatory pathways and act as energy substrates for metabolically active tissues [13,26,27].

The comparison between dietary regimens highlights clear distinctions: KD elicited the greatest improvements across all variables, while 16:8 fasting showed similarly beneficial but slightly less pronounced effects. The 5:2 fasting regimen displayed weaker metabolic responses and minimal orexinergic activation. These differences suggest that the consistency and pattern of nutrient timing may play a crucial role in neuro-metabolic adaptation [28]. Time-restricted feeding enforces stable fasting cycles, potentially enhancing circadian alignment of orexinergic neurons, while KD sustains ketone-driven metabolic signaling that may synergize with orexin pathways. The minimal effects observed with the 5:2 model suggest that intermittent caloric restriction without sustained fasting or ketosis may be insufficient to robustly activate orexinergic circuits [29].

Collectively, these findings underscore the role of Orexin-A as a central modulator linking dietary patterns, metabolic flexibility, and inflammation. The strong correlations between Orexin-A and improvements in adiposity, glycemia, lipid profile, and lean mass point toward a broader framework in which orexinergic signaling serves as a key component of adaptive metabolic remodeling. Orexin-A is widely recognized as a key regulator of energy expenditure and metabolic activation. Beyond its central role in maintaining wakefulness, Orexin-A stimulates sympathetic nervous system output, promotes lipid oxidation, enhances brown adipose tissue thermogenesis, and increases spontaneous physical activity—mechanisms that collectively contribute to higher daily energy expenditure [30,31,32,33,34]. Experimental studies have demonstrated that orexinergic signaling increases mitochondrial respiration, improves substrate switching, and facilitates metabolic flexibility, allowing efficient transitions between carbohydrate and lipid metabolism [35,36,37,38]. Obesity is typically associated with reduced orexinergic tone, partly due to chronic inflammation, which suppresses orexin neuron activity through cytokine-mediated inhibitory pathways [39,40,41]. Therefore, the progressive rise in Orexin-A observed during the intervention may reflect not only a direct neuroendocrine adaptation to dietary patterns but also a release from inflammatory inhibition. This restoration of orexinergic activation is consistent with improvements in metabolic flexibility, increased lipid utilization, and enhanced energy expenditure. These mechanisms may partially explain the favorable metabolic outcomes observed in the intervention groups, particularly in protocols that promote stable circadian entrainment and reduced inflammatory load [31,42]. Although mediation analyses suggested that Orexin-A may partially explain some metabolic changes, causality cannot be inferred. Only experimental or pharmacological manipulation of the orexinergic system could definitively establish a causal pathway.

This supports the hypothesis that enhancing orexinergic pathways—either through nutritional strategies or potential pharmacological approaches—could represent a promising direction in obesity management [43,44].

This study has some limitations. The sample size was relatively small (n = 30), although comparable to similar nutritional intervention studies. Dietary adherence, although monitored, may still be subject to self-reporting bias. The ketogenic group followed a low-calorie ketogenic protocol, which may limit direct comparison with isocaloric ketogenic diets. Finally, the absence of a non-intervention control group prevents isolating the absolute effect of each dietary strategy. The present study provides evidence that both the Ketogenic Diet and 16:8 Intermittent Fasting induce substantial metabolic and neuroendocrine benefits in obese individuals, largely mediated through increases in Orexin-A. These findings highlight the potential of incorporating orexin-based biomarkers into clinical evaluation of dietary interventions and reinforce the role of targeted nutritional strategies for improving metabolic health. Nevertheless, this study has several limitations: small sample size, single-center design, absence of blinding, differences in caloric restriction across interventions, reliance on self-reported dietary adherence, lack of detailed assessment of sleep and physical activity, and the exploratory nature of mediation analyses. Strengths include the 12-month duration, randomized three-arm design, and combined evaluation of metabolic, inflammatory, and neuroendocrine markers.

## 5. Conclusions

This study demonstrates that both the Ketogenic Diet and 16:8 Intermittent Fasting produce substantial improvements in body composition, metabolic biomarkers, and inflammatory status in obese individuals. These benefits were strongly associated with increases in circulating Orexin-A, highlighting its potential role as a mediator of neuro-metabolic adaptation. The findings suggest that the Orexin-A correlates inversely with BMI, fat mass, fasting glucose, and total cholesterol, supporting its role in metabolic flexibility. In addition, KD and 16:8 fasting are effective, sustainable interventions capable of enhancing orexinergic activity. The 5:2 regimen shows weaker metabolic and neuroendocrine effects, indicating that consistency and metabolic pathway engagement are key drivers of adaptation. Orexin-A emerges as a promising biomarker to assess the neuroendocrine impact of dietary interventions and may represent a valuable tool in personalized nutrition for obesity management. In conclusion, the ketogenic diet produced the largest and most sustained metabolic improvements, while the 16:8 fasting protocol yielded rapid early benefits and a consistent neuroendocrine response. These findings are preliminary and should be interpreted cautiously due to the small sample size. Further large-scale randomized trials are required to confirm these observations.

Several limitations of the present study should be acknowledged. First, although adherence to the dietary interventions was closely monitored through structured dietary logs and periodic recalls, nutritional ketosis in the ketogenic diet group was not objectively verified using biochemical measurements (e.g., circulating β-hydroxybutyrate or urinary ketone assessments). As a result, the degree and consistency of ketosis across participants cannot be directly quantified, and adherence relied primarily on self-reported dietary data, which may be subject to reporting bias.

Second, the relatively small sample size and the inclusion of a restricted age range may limit the generalizability of the findings to broader populations with different demographic or clinical characteristics. While randomization ensured baseline comparability among groups, residual inter-individual variability cannot be fully excluded.

Third, the study involved the assessment of multiple anthropometric, metabolic, inflammatory, and neuroendocrine outcomes, increasing the potential risk of type I error due to multiple testing. Although within-outcome post hoc comparisons were adjusted and a hierarchical interpretation of primary versus secondary endpoints was applied, findings related to secondary and exploratory outcomes should be interpreted with caution.

Fourth, Orexin-A was investigated as an exploratory biomarker, and its circulating levels may not fully reflect central orexinergic activity. Therefore, mechanistic inferences regarding central neuroendocrine regulation should be considered preliminary and hypothesis-generating.

Finally, although sensitivity analyses adjusted for age and sex were performed and confirmed the robustness of the primary findings, the observational nature of biomarker associations and mediation analyses precludes definitive causal conclusions. Future studies incorporating larger samples and direct assessments of central neuroendocrine activity are warranted to further elucidate the mechanisms underlying dietary modulation of metabolic health.

## Figures and Tables

**Table 1:** Baseline (T0) anthropometric, metabolic, and neuroendocrine characteristics by dietary intervention group.

Outcome (T0)	KD (*n* = 10)	TRF 16:8 (*n* = 10)	ADF 5:2 (*n* = 10)	Between-Group *p* at T0
BMI (kg/m^2^)	33.4 ± 2.9	33.7 ± 3.0	34.3 ± 3.2	*p* = 0.43
Fat mass (%)	34.7 ± 3.1	36.2 ± 3.3	38.1 ± 3.5	*p* = 0.35
Total cholesterol (mg/dL)	252 ± 23.2	250 ± 24.1	251 ± 25.9	*p* = 0.41
Fasting glucose (mg/dL)	108 ± 8.3	110 ± 9.6	112 ± 9.3	*p* = 0.39
Orexin-A (ng/mL)	2.1 ± 0.3	2.0 ± 0.3	2.1 ± 0.3	*p* = 0.47

Baseline values of body mass index (BMI), fat mass, fasting glucose, total cholesterol, and circulating Orexin-A in participants assigned to the ketogenic diet (KD), time-restricted feeding 16:8 (TRF 16:8), and alternate-day fasting 5:2 (ADF 5:2). Data are presented as mean ± standard deviation (SD). Between-group comparisons at baseline (T0) were performed using one-way analysis of variance (ANOVA). No statistically significant differences were observed among groups at baseline, indicating comparable anthropometric, metabolic, and neuroendocrine profiles prior to the intervention.

**Table 2:** Between-group pairwise comparisons at each time point for anthropometric, metabolic, and neuroendocrine outcomes (Tukey-adjusted *p*-values).

Outcome	Time	KD vs. TRF 16:8	KD vs. ADF 5:2	TRF 16:8 vs. ADF 5:2
**BMI (Figure 1)**	T0	n.s.	n.s.	n.s.
	T1	n.s.	n.s.	n.s.
	T2	n.s.	n.s.	n.s.
	T3	*p* = 0.11	*p* = 0.004	n.s.
**Fat mass (Figure 2)**	T0	n.s.	n.s.	n.s.
	T1	n.s.	n.s.	n.s.
	T2	n.s.	n.s.	n.s.
	T3	*p* = 0.14	*p* = 0.006	n.s.
**Total cholesterol (Figure 3)**	T0	n.s.	n.s.	n.s.
	T1	n.s.	n.s.	n.s.
	T2	n.s.	n.s.	n.s.
	T3	n.s.	*p* = 0.008	n.s.
**Fasting glucose (Figure 4)**	T0	n.s.	n.s.	n.s.
	T1	n.s.	n.s.	n.s.
	T2	n.s.	n.s.	n.s.
	T3	n.s.	*p* = 0.007	n.s.
**Orexin-A (Figure 5)**	T0	n.s.	n.s.	n.s.
	T1	n.s.	n.s.	n.s.
	T2	n.s.	n.s.	n.s.
	T3	n.s.	*p* = 0.006	n.s.

Pairwise between-group comparisons were performed using Tukey’s honestly significant difference (HSD) post hoc tests following two-way repeated-measures ANOVA (time × group). Reported values are Tukey-adjusted *p* values. n.s., not significant (*p* ≥ 0.05). T0–T3 indicate baseline and follow-up time points; T3 corresponds to the end of the intervention.

**Table 3:** Main effects of time and time × group interactions from two-way repeated-measures ANOVA on anthropometric, biochemical, and hormonal parameters.

Parameter	Main Time Effect	Interaction (Time × Group)	η^2^	R^2^	Strongest Response
BMI	*F* = 12.46, *p* < 0.001	*F* = 4.37, *p* = 0.002	0.42	0.51	Ketogenic diet
Fat Mass	*F* = 14.28, *p* < 0.001	*F* = 5.12, *p* = 0.001	0.46	0.49	Ketogenic diet
Lean Mass	*F* = 10.97, *p* < 0.001	*F* = 4.05, *p* = 0.002	0.41	0.46	Ketogenic diet
Total Cholesterol	*F* = 13.35, *p* < 0.001	*F* = 4.76, *p* = 0.001	0.44	0.48	Ketogenic diet
Glucose	*F* = 15.02, *p* < 0.001	*F* = 5.11, *p* < 0.001	0.47	0.50	Ketogenic diet
Orexin-A	*F* = 16.84, *p* < 0.001	*F* = 4.94, *p* < 0.001	0.50	0.52	Ketogenic diet

Results of the two-way repeated-measures ANOVA assessing the main effects of time and the time × group interaction on anthropometric, biochemical, and hormonal parameters. Values represent F statistics, significance levels (*p*), and effect sizes (partial η^2^). R^2^ values reflect the proportion of explained variance. The Ketogenic diet (KD) consistently elicited the strongest adaptive response across all measured outcomes.

**Table 4:** Baseline (T0), end-of-study (T3), and changes from baseline (ΔT3 − T0) in anthropometric, metabolic, and neuroendocrine outcomes.

Outcome	Time	KD (n = 10)	TRF 16:8 (n = 10)	ADF 5:2 (n = 10)
**BMI (kg/m^2^)**	T0	33.4 ± 2.9	33.7 ± 3.0	34.3 ± 3.2
	T3	29.1 ± 2.5	30.4 ± 2.6	33.5 ± 3.0
	Δ(T3 − T0)	−4.3	−3.3	−0.8
**Fat mass (%)**	T0	34.7 ± 3.1	36.2 ± 3.3	38.1 ± 3.5
	T3	28.9 ± 2.7	31.6 ± 2.9	35.8 ± 3.1
	Δ(T3 − T0)	−5.8	−4.6	−2.3
**Total cholesterol (mg/dL)**	T0	252 ± 23	250 ± 24	251 ± 25
	T3	196 ± 18	205 ± 19	230 ± 22
	Δ(T3 − T0)	−56	−45	−21
**Fasting glucose (mg/dL)**	T0	108 ± 8	110 ± 9	112 ± 9
	T3	87 ± 6	95 ± 7	106 ± 8
	Δ(T3 − T0)	−21	−15	−6
**Orexin-A (ng/mL)**	T0	2.1 ± 0.3	2.0 ± 0.3	2.1 ± 0.3
	T3	3.4 ± 0.4	3.0 ± 0.4	2.6 ± 0.3
	Δ(T3 − T0)	+1.3	+1.0	+0.5

Baseline (T0) and end-of-study (T3) values, together with absolute changes from baseline (ΔT3 − T0), for body mass index (BMI), fat mass, fasting glucose, total cholesterol, and circulating Orexin-A in participants assigned to the ketogenic diet (KD), time-restricted feeding 16:8 (TRF 16:8), and alternate-day fasting 5:2 (ADF 5:2). Data are presented as mean ± standard deviation (SD). T3 corresponds to the end of the 12-month intervention period.

## Data Availability

The raw data supporting the conclusions of this article will be made available by the authors on request.
